# Differential Methylation and Transcriptome Integration Analysis Identified Differential Methylation Annotation Genes and Functional Research Related to Hair Follicle Development in Sheep

**DOI:** 10.3389/fgene.2021.735827

**Published:** 2021-09-30

**Authors:** Yuezhen Tian, Xuemei Yang, Jianwen Du, Weidan Zeng, Weiwei Wu, Jiang Di, Xixia Huang, Kechuan Tian

**Affiliations:** ^1^The Key Laboratory for Genetics Breeding and Reproduction of Xinjiang Cashmere and Wool Sheep, Institute of Animal Science, Xinjiang Academy of Animal Sciences, Urumqi, China; ^2^College of Animal Science, Xinjiang Agricultural University, Urumqi, China

**Keywords:** sheep, hair follicle development, DNA methylation, MeDIP-seq, fibroblast, WNT2

## Abstract

Hair follicle growth and development are a complex and long-term physiological process, which is regulated by a variety of physical factors and signal pathways. Increasing the understanding of the epigenetic regulation and function of candidate genes related to hair follicle development will help to better understand the molecular regulatory mechanisms of hair follicle development. In this study, the methylated DNA immunoprecipitation sequencing (MeDIP-seq) was used to obtain the genome-wide methylation map of the hair follicular development of Super Merino sheep in six stages (fetal skin tissue at 65d, 85d, 105d, 135d, 7d, and 30d after birth). Combined with the results of previous RNA-sequencing, 65 genes were screened out that were both differential methylation and differential expression, including EDN1, LAMC2, NR1D1, RORB, MyOZ3, and WNT2 gene. Differential methylation genes were enriched in Wnt, TNF, TGF-beta, and other signaling pathways related to hair follicle development. The bisulfite sequencing PCR results and MeDIP-seq were basically consistent, indicating that the sequencing results were accurate. As a key gene in the Wnt signaling pathway, both differential methylation and expression gene identified by MeDIP-seq and RNA-seq, further exploration of the function of WNT2 gene revealed that the DNA methylation of exon 5 (CpG11 site) promoted the expression of WNT2 gene. The overexpression vector of lentivirus pLEX-MCS-WNT2 was constructed, and WNT2 gene effectively promoted the proliferation of sheep skin fibroblasts. The results showed that WNT2 gene could promote the growth and development of skin and hair follicles. The results of this study will provide a theoretical basis for further research on sheep hair follicle development and gene regulation mechanisms.

## Introduction

With the development of textile industry, people’s demand for high-quality wool is increasing, and China has been relying on imported wool to meet the demand of domestic production for a long time. In order to break the foreign monopoly on the ultra-fine wool sheep market, improve the grade and level of the fine wool sheep industry in China, and protect the diversity of sheep breeds, it is of great significance to select and improve the fine wool sheep breeds in China to meet the market demand. Super Merino sheep is a new breed of ultra-fine wool independently bred in China. This breed has been jointly bred by several units in Xinjiang, Inner Mongolia, Jilin and other provinces since 2000. With ultra-fine Australian Merino sheep as the male parent, and Chinese Merino sheep Xinji fine wool sheep and Aohan fine wool sheep as the female parent, the method of progressive hybridization has been adopted. After 14years of selection and breeding, the new breed of ultra-fine wool for worsted with wool fineness of 17.0~19.0μm was approved and named in 2014. However, the regulation mechanism of wool growth and hair follicle development is still unclear. Therefore, it is necessary to further breed Super Merino sheep as an excellent local ultra-fine wool breed in China and to explore the mechanism related to the development of its hair follicles.

Hair is the product of hair follicle growth and development, and hair follicle is the skin of the derivatives, its structure and properties have important effects on yield and quality of hair (Cao et al., 2017; Zhao et al., 2020a), so increasing related studies on hair follicle is necessary. Hair follicle growth and development are a complex and long-term physiological process regulated by the variety of physical factors and signaling pathways, and gene expression is one of the decisive factors influencing the growth of sheep hair (Sulayman et al., 2019; Zhang et al., 2019a). DNA methylation is an important epigenetic mechanism that plays a role in several biological processes, such as gene expression and genomic imprinting (Freitag and Selker, 2005; Devos et al., 2021), and also plays a role by regulating the expression of genes related to hair follicle development (Zou et al., 2016; Wang et al., 2020; Li et al., 2021). At present, researchers have obtained the transcriptome regulation network of hair follicle development in some varieties of sheep through multi-omics (Sulayman et al., 2019; Li et al., 2020), but few epigenetic markers related to it have been found. Therefore, further studies are needed to integrate the results of multi-omics studies, in order to better analyze the regulation mechanism of hair follicle development.

Methylated DNA immunoprecipitation sequencing (MeDIP-seq) is a high-throughput sequencing technology based on the principle of antibody enrichment, which specifically binds 5mC bound antibodies to DNA methylation fragments on the genome, and then enriches and rebinds specifically (Zhou et al., 2018; Ben Maamar et al., 2021). This method has high specificity and is convenient and suitable for DNA methylation analysis. Compared with the whole-genome bisulfate sequencing method, the cost is lower and the genome-wide methylation status can be estimated unbiased. Therefore, this method was used in this study to analyze the genome-wide methylation level (Willems et al., 2016; Kim et al., 2018).

In order to explore the potential regulatory relationships among epigenetic regulation, gene expression, and gene function, this study will continue to explore regulation mechanism of genes that are both differential methylation and differential expression in different hair follicle stages (Sulayman et al., 2019), including differential methylation regions (DMRs) and differential methylation genes (DMGs; Chen et al., 2018a; Zhang et al., 2019b). As the encoding gene of methyltransferase, DNMT1, DNMT3a, and DNMT3b are involved in the regulation of target gene methylation (Yu et al., 2021). Therefore, the expression levels of DNMTs genes in different hair follicle development stages will be explored in this study. Then, WNT2 is a differential methylation and differential expression gene in different stages of hair follicle development, and WNT2 is also a key gene in the Wnt signaling pathway related to hair follicle development (Fu and Hsu, 2013; Chen et al., 2018a; Zhou et al., 2020). Therefore, we chose WNT2 gene to further explore its regulation and functional mechanism of methylation in hair follicle development. Firstly, we will explore the DNA methylation, mRNA, and protein expression level of exon 5 of WNT2 gene, and analyze the relationship between DNA methylation and expression level of this gene. Secondly, lentivirus PLEX-MCS-WNT2 expression vector will be constructed, and the function of WNT2 gene will be preliminarily verified. And then, the lentivirus pLEX-MCS-WNT2 expression vector will be co-transfected into sheep skin fibroblasts. Finally, the proliferation of sheep skin fibroblasts can be identified by CCK-8 method to further determine the effect of WNT2 gene on the development of hair follicles. The results of this study will provide a theoretical basis for the subsequent research on the development of hair follicles and the mechanism of gene regulation in sheep hair. It is of great significance to explain the regulatory network of hair follicle development from the perspective of molecular and epigenetic mechanisms.

## Materials and Methods

### Samples Collection and Cell Culture

All Super Merino sheep used in this study were raised in the Xinjiang Science and Innovation Breeding Center (Latitude 43°01′08″–44°06′11″N, Longitude 86°37′56″–88°58′22″E), Xinjiang Uygur Autonomous Region, China. All selected sheep were pluriparous sheep about 2 to 3years old. The ewes were in estrus at the same time and were mated with the semen of the same ram and were managed under the same environmental and nutritional conditions. In this study, fetal skin tissue at 65d, 85d, 105d, and 135d at embryonic period and 7d and 30d after birth (labeled as G1, G2, G3, G4, G5, and G6, respectively) were collected, and three lambs were collected at each stage (Sulayman et al., 2019). After collection and treatment, the samples were quickly placed into a cryopreservation tube and stored in a liquid nitrogen. In addition, five ewes with an average fiber diameter of 16.9μm (extremely fine group) and five ewes with an average fiber diameter of 19.53μm (extremely thick group) were selected. Skin tissue samples (2cm×2cm) from the left scapula were collected and cryopreserved in liquid nitrogen for DNA, RNA, and protein extraction for further WNT2 gene function verification. Experiments were performed according to the Regulations for the Administration of Affairs Concerning Experimental Animals and approved by the Xinjiang Agricultural University.

The sheep skin fibroblasts used in this experiment were donated by the Biotechnology Institute of Xinjiang Academy of Animal Science. The cells were seeded in a petri dish containing 20% fetal bovine serum and 1% antibiotics in 5% CO_2_ at 37°C.

### Genomic DNA, RNA, and Protein Extraction

The AllPrep DNA/RNA Mini Kit (80204, Qiagen) was used to extract genomic DNA from skin tissue of Super Merino sheep at six stages of hair follicle development: 65d, primary hair follicles formation; 85d, secondary hair follicle bud formation; 105d, differentiation of secondary hair follicles; 135d, secondary hair follicles mature; and 7d and 30d, after birth (Ablat et al., 2017). The extracted genomic DNA was subjected to NanoDrop One spectrophotometer and Qubit 2.0 (Q33216, Invitrogen) electrophoresis for qualified quality inspection and further experiment.

Tissues RNA was extracted by the AllPrep DNA/RNA Mini Kit (80204, Qiagen), and RNA concentration was determined using NanoDrop 2000 nucleic acid detector. After agarose gel electrophoresis, cDNA was synthesized using reverse transcription kit (PrimeScript RT reagent Kit with gDNA Eraser) and stored for further experiment in the −20°C refrigerator.

Tissue samples of 50–100mg were triturated in liquid nitrogen, and protein samples were extracted from the skin tissues of the 10 sheep individuals (five extremely fine group and five extremely thick group sheep) according to the instructions of highly efficient RIPA tissue/cell rapid lysate. The extracted protein samples were detected according to the instructions of the Pierce TM BCA Protein Assay Kit, and the qualified protein samples were stored at −20°C.

### MeDIP-seq Analysis

DNA libraries were constructed as: G1, G2, G3, G4, G5, and G6. MeDIP-seq was performed as previously described by previous method (Miao et al., 2020). Purified DNA was analyzed on NanoDrop One spectrophotometer (Thermo Fisher Scientific, Waltham, MA, United States) and quantified using a Qubit (Thermo Fisher Scientific, Waltham, MA, United States). DNA was recovered with AMPure XP beads (Beckman Coulter, Inc., Indianapolis, IN, United States) and utilized for MeDIP using a Magnetic Methylated DNA Immunoprecipitation Kit (Diagenode Inc., Denville, NJ, United States) according to the manufacturer’s protocol. DNA size was verified using an Agilent DNA-1000 Kit on an Agilent 2100 Bioanalyzer (Agilent Technologies, Santa Clara, CA, United States). After MeDIP, the remaining DNA was PCR-amplified with sequencing primers and the DNA libraries were quantified using a Qubit fluorometer (Thermo Fisher Scientific, Waltham, MA, United States) according to the Qubit user guide, and subsequently used for sequencing (Illumina Hiseq2500, Illumina). The raw data were aligned to the *Ovis aries* genome (version 0.12.8) using default parameters (Li et al., 2021). The number of reads was assessed using the chi-squared test and false discovery rate; results with *p*<0.05 were considered significant. MACS (version 1.4.2) software was applied to search the peak enrichment area, and the enrichment area of the sample peak was obtained.

According to the previous RNA-seq results of our research group (Sulayman et al., 2019), the tissues of the animals used were the same as that of the MeDIP-seq. According to the results of the two analysis, genes/regions that were both differentially methylated and differentially expressed were screened to provide a basis for subsequent analysis.

### GO and KEGG Pathway Analysis

DMGs were subjected to GO functional analysis and KEGG pathway analysis by DAVID. The GO enrichment analysis provided all significantly enriched GO terms for comparison with the DMGs to determine the genomic background and filters of the DMGs that corresponded to biological functions. Pathway analysis for the DMGs was performed using the KEGG tool, which is a digital representation of the biological system (Kanehisa et al., 2006). GO enrichment analysis for the DMGs was performed using the GOseq R package (Young et al., 2010). GO enrichment can classify DMG according to biological processes, cell components, molecular functions, etc. KEGG pathway can also be significantly enriched by the GO enrichment principle, and corrected values of *p*<0.05 were considered significantly enriched by the DMGs.

### Bisulfite Sequencing PCR

The differential methylation and differentially expressed genes related to the regulation of hair follicle development in 105d and 135d of embryonic period were selected for bisulfite sequencing PCR (BSP) analysis. Genomic DNA was processed according to the instructions of the EZ DNA Methylation-GOIDTM Kit. We downloaded the sequences of *Ovis aries* EDN1, LAMC2, NR1D1, RORB, MYOZ3, and WNT2 gene from NCBI database and designed the primer pairs of BSP by MethPrimer online software. The modified DNA was amplified by PCR, as previously reported (Fan et al., 2020). PCR amplification product was purified by the PCR product purification kit (Tiangen). After agarose gel electrophoresis and concentration determination, the purified product was linked to PMD19-T vector (Takara). Three positive clones from each sample were selected and sent to Shanghai Sangong for sequencing.

### Expression Analysis of DNMTs, EDN1, and WNT2 Genes in Sheep

Fetal skin tissue at 65d, 85d, 105d, 135d, and 7d and 30d after birth (labeled as G1, G2, G3, G4, G5, and G6, respectively) were used, and three samples were collected at each stage. According to the sequences of sheep DNMT1, DNMT3a, DNMT3b, and EDN1 provided by the NCBI, the reference gene was GAPDH, and Primer Premier 5.0 software was used to design the quantitative primers. The cDNA template was used for qRT-PCR amplification. The total system was 25μl, including 1μl cDNA, and each sample had three replicates. The amplification reaction was carried out in Bio-Rad CFX96 fluorescent quantitative PCR instrument.

The gene expressions of WNT2 of the skin, heart, liver, spleen, lung, kidney, and muscle tissue of left shoulder of the lambs at 135d (hair follicle maturation period) were identified. According to the sequences of sheep WNT2, the reference gene was GAPDH (Kang et al., 2020). The amplification procedures and systems were same as above.

### Construction of Lentivirus pLEX-MCS-WNT2 Expression Vector

The restriction endonuclease *XhoI* and *NotI* were used to digest the WNT2 fragment and the vector by double enzyme digestion. Restrictive endonuclease NotI and XhoI restriction sites and protective bases were introduced. Forward primers: aacgagatatcgggtcccGCGGCCGCATGAACGCCT, reverse primers: tgtgttctcgtcgtgcccCTCGAGTCATGTCGGAGCCG. The digested product was recovered according to the agarose gel DNA recovery kit. The recovered product was linked to the pLEX-MCS vector, and the ligated product was added to the DH5α capable cells. The correct bacterial colonies were selected for expanded culture. Meanwhile, the recombinant plasmid was extracted using the instructions of Tiangen endotoxin-free plasmid DNA small extraction kit.

### Transfection of Recombinant Plasmid and Cell Proliferation Assay

Sheep skin fibroblasts were evenly inoculated into 24-well plates with 1×10^5^ cells per well and cultured in a CO_2_ incubator. When the confluence degree of sheep skin fibroblasts reached 70–80%, the co-infection test of the recombinant plasmid was carried out (Saadeldin et al., 2020). Each sample was repeated with six Wells. The transfection test groups were blank group (no treatment), control group (only lentivirus), and experimental group (lentivirus and WNT2).

After transfection for 48h, sheep skin fibroblasts were digested with trypsin and added to the basic medium to stop digestion. 1ml DMEM was added to each well and inoculated to 96-well plate at 100μl/well and given 10μl CCK-8 solution. After treatment for 0, 24h, 48h, and 72h, the absorbance at 450nm was measured with a microplate analyzer. The OD value represented the relative growth level of sheep fibroblasts, and the cell proliferation curve was plotted.

### Statistical Analysis

The relative expression level of the target gene was calculated using the method of 2^-△△Ct^. The SPSS19.0 software was used for one-way ANOVA and independent sample t test, and Duncan method was used for multiple comparisons. *p*<0.05 indicated significant difference, and *p*<0.01 indicated significant difference. The relative expression level of WNT2 protein in the skin tissues of Sub-Merino sheep with different fineness was determined by ImageJ. Data were presented as mean±SEM.

## Results

### Global Mapping of DNA Methylation in Skin Tissue of Six Stages

**Figure 1:**
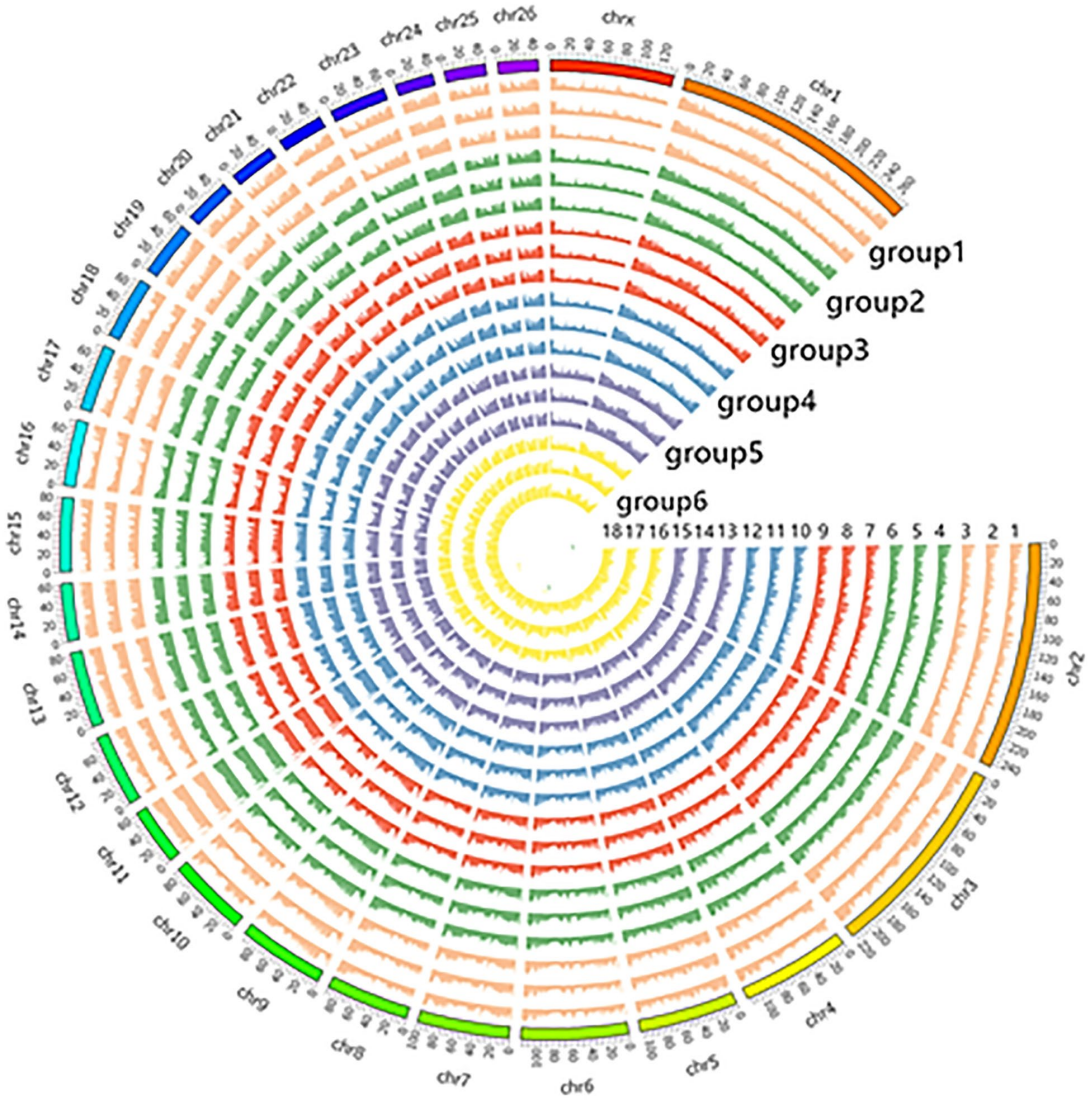
Display of circos in genome-wide methylation levels.

**Figure 2:**
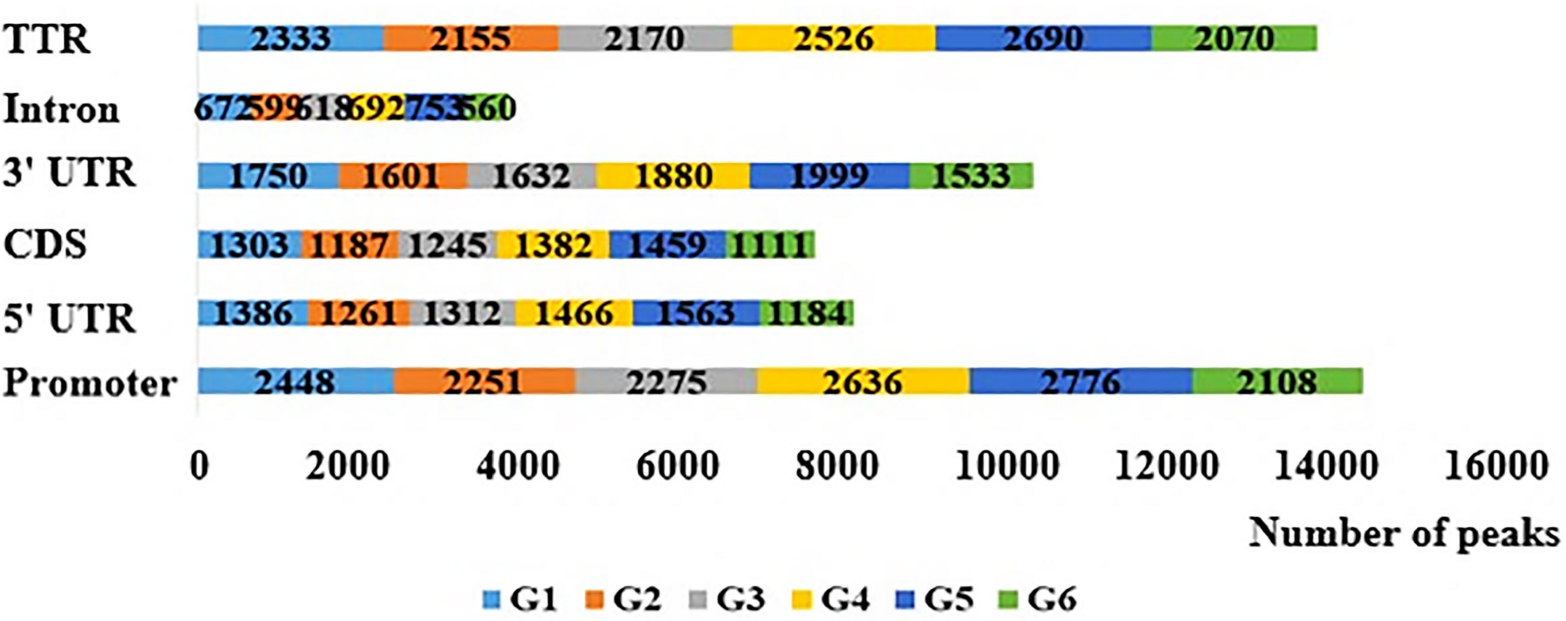
Distribution of peaks in different gene elements in the six stage. Promoter is 2,000bp upstream of transcription start site and TTR is 5,000bp downstream of transcription stop site.

The number of original reads provided by each sample after sequencing was about 6G, and the proportion of base mass greater than 20 (Q20) per direction was more than 94%, which could be used for subsequent analysis (Table 1). Illumina NovaSeq was used to analyze the MeDIP-seq of skin tissues of 18 samples from six periods of Super Merino sheep, and the Clean ratio was between 97.64 and 98.73%. The obtained clean reads were compared with the reference genome of *Ovis aries* by Bowtie software, and the only matched reads were used for subsequent analysis. The circos graph of genome-wide DNA methylation level was drawn using circos software, with the length of 100kb as the window. Both autosomes (1–26) and sex chromosomes (X) are rich in sequence enrichment peaks (Figure 1). The number of Peak scanned in individuals range from 75,514 to 129,618, and most of them are distributed in the intergenic region and promoter region (Figure 2). The raw sequencing data has been uploaded to the NCBI database (https://www.ncbi.nlm.nih.gov/).

**Table 1:** A statistics of data preprocessing results.

Sample No	Raw reads	Quality trimed	Adaptor trimed	Clean reads	Clean ratio(%)
No.1 (G1-1)	53,845,124	495,554	47,925	53,115,802	98.65%
No.2 (G1-2)	54,982,106	503,671	48,811	54,242,600	98.66%
No.3 (G1-3)	57,072,144	514,604	50,548	56,303,779	98.65%
No.4 (G1-1)	63,036,942	564,459	56,251	62,222,198	98.71%
No.5 (G1-2)	57,441,046	518,801	51,159	56,654,443	98.63%
No.6 (G1-3)	51,338,890	465,642	46,059	50,675,433	98.71%
No.7 (G1-1)	52,919,506	473,483	47,374	51,807,185	97.90%
No.8 (G1-2)	60,987,212	561,587	55,349	60,181,362	98.68%
No.9 (G1-3)	54,714,588	485,909	49,407	54,014,211	98.72%
No.10 (G1-1)	66,545,134	592,933	59,103	65,681,906	98.70%
No.11 (G1-2)	58,959,386	540,447	54,116	58,142,509	98.61%
No.12 (G1-3)	63,980,500	576,058	54,460	63,147,351	98.70%
No.13 (G1-1)	69,863,230	637,525	61,321	68,941,380	98.68%
No.14 (G1-2)	59,941,976	516,709	53,274	59,179,417	98.73%
No.15 (G1-3)	78,318,998	711,762	69,293	77,307,653	98.71%
No.16 (G1-1)	58,779,108	530,707	52,018	58,022,063	98.71%
No.17 (G1-2)	41,052,114	365,460	35,157	40,082,620	97.64%
No.18 (G1-3)	53,542,530	486,401	47,371	52,857,557	98.72%

*Effective reads ratio=Clean reads/raw reads*.

### MeDIP-seq and RNA-seq Combination Analysis

**Figure 3:**
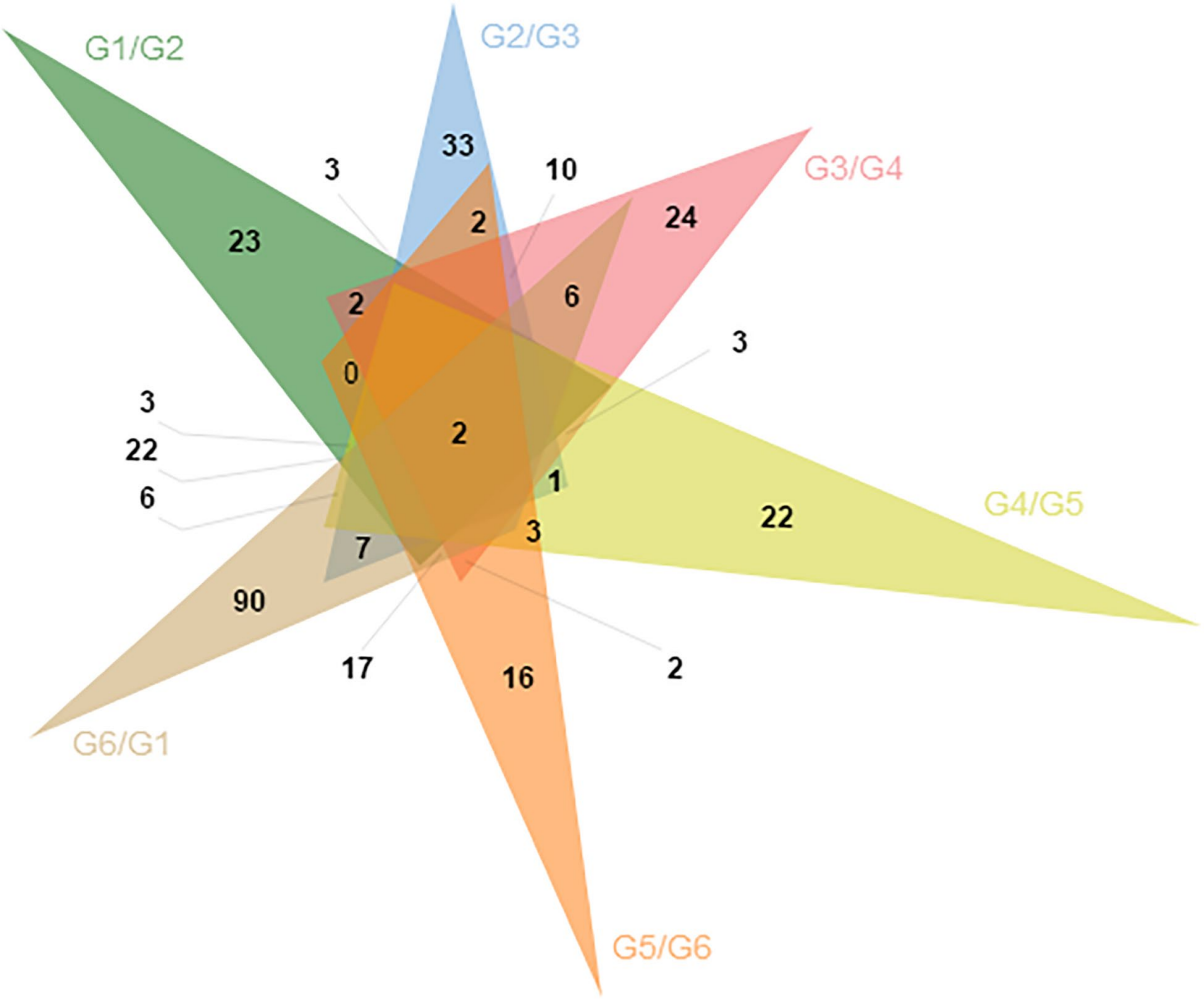
The Venn of differential methylation genes in the six group comparisons.

Through differential methylation analysis of two adjacent periods, a total of 596 differentially methylated genes were obtained, among which 256 were up-methylated genes and 340 were down-methylated genes. The down-methylated genes were more than up-methylated genes. There were 93 differentially methylated genes in the G1/G2 group, 89 differentially methylated genes in the G2/G3 group, 76 differentially methylated genes in the G3/G4 group, 64 differentially methylated genes in the G4/G5 group, 69 differentially methylated genes in the G5/G6 group, and 205 differentially methylated genes in the G6/G1 group. Two of the DMGs were present in all six comparison groups (Figure 3).

DNA methylation will affect gene expression to some extent, so the 596 differentially methylated genes screened in this study were overlapped with the differentially expressed genes previously screened by our group, and a total of 65 both differentially methylated and differentially expressed genes were identified in two adjacent periods (Table 2).

**Table 2:** Genes obtained from unite analysis of methylated DNA immunoprecipitation sequencing (MeDIP-Seq) and RNA-Seq.

Stages	Genes	Gene counts
G1/G2	*LAMC2, ARHGAP36, PADI3, NR1D1, EDN1*	5
G2/G3	*GABRB2, FABP3, B2M, PLIN5, CAPN3, K38, SELE, SLC5A1, MAPK13, CDKN1A*	10
G3/G4	*MYOZ3, MX1, RORB, WNT2, SLC5A1, EDN1, SLC14A1*	7
G5/G6	None	0
G6/G1	*SFN, AK1, LPIN1, AQP5, ACP5, DCT, NR1D1, RHOF, PQLC1, G6PD, ARHGAP36, GSN, SFTPC, TUBA4A, ITM2C, AZIN2, PADI3, B4GALT7, IGF1, IFT27, TSPAN5, TOB2, MYOZ3, SCD5, MMP14, B2M, SDR16C5, ATP7B, ACACA, MC1R, TIMP2, CYGB, AVPR1B, TLR5, SELP, LAMC2, IL2RA, REM1, HSD17B2, FURIN, SERPINA1, CRYAB, ARNTL, GHR, EDN1, NFKBIA, GNAI2, SLC25A27, AQP4, FHL1*	50

### Gene Ontology and Pathway Enrichment Analysis

**Figure 4:**
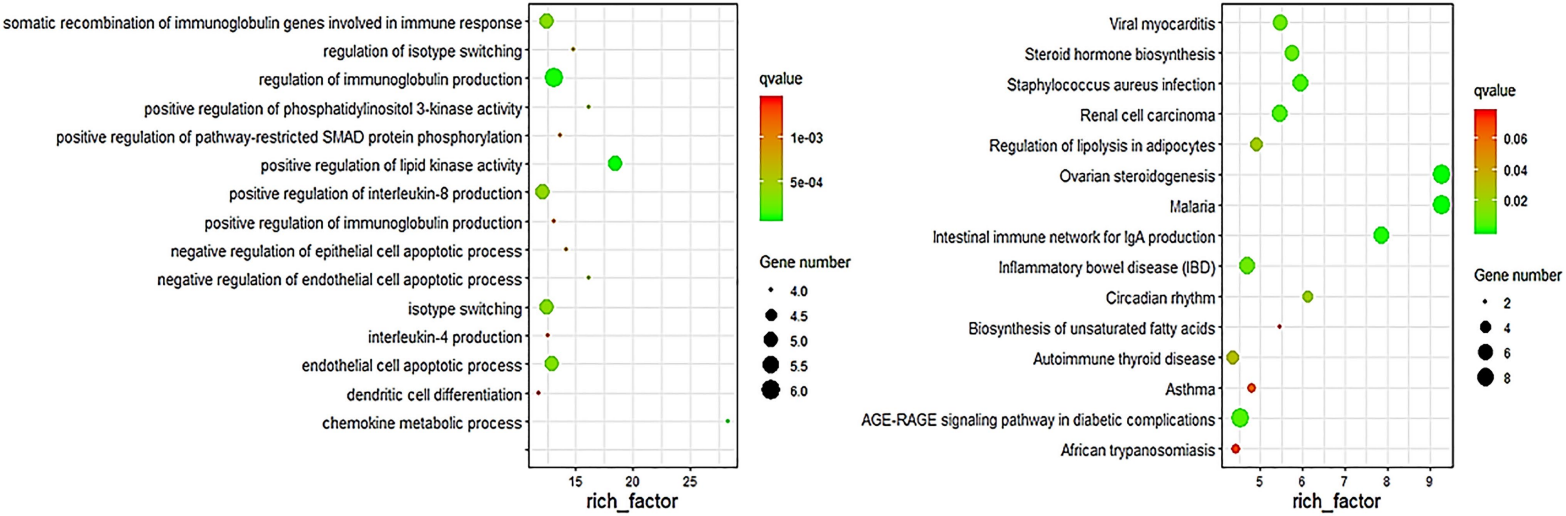
Top 15 biological processes of GO enrichment and pathway enrichment.

In order to assess whether the genes associated with differential methylation were enriched in certain biological processes or pathways, we conducted gene ontology and pathway analyses using the DMGs. DAVID was used to perform GO and KEGG enrichment analysis on the differentially methylated genes obtained at six stages to screen the biological functions and signaling pathways related to the hair follicle development of Super Merino sheep. The GO functional annotation showed that 1,046 GO entries were enriched, including 76 entries for cellular components, 124 entries for molecular functions, and 846 entries for biological processes. The enrichment of biological processes was mainly due to the regulatory effects of the generation of immune-related products, chemokines, enzyme activities, and apoptosis processes of epithelial and endothelial cells. Go items related to hair follicle development: hair follicle development, hair cycle process, skin epidermis development, and cell differentiation (Figure 4).

KEGG enrichment analysis showed that differentially methylated genes were enriched in 220 signaling pathways, and Top 15 was selected according to the enrichment degree. Among them, metabolic pathways contained the most differentially methylated genes, and these genes were enriched in WNT signaling pathway, TNF signaling pathway, MAPK signaling pathway, TGF-beta signaling pathway, ECM-receptor interaction, and other signaling pathways related to hair follicle development (Figure 4).

### Differential Methylation Genes Related to Hair Follicle Development in Super Merino Sheep

Bisulfite sequencing PCR primers of EDN1, LAMC2, NR1D1, RORB, MYOZ3, and WNT2 genes were used for PCR amplification using bisulfite modified DNA as template (Table 3). The target fragment in the DMR region of EDN1 gene was 279–551bp, the product size was 273bp, there were 10 methylation sites, and the GC content ranged from 50 to 56%. The target fragment in the DMR region of LAMC2 gene was 198–497bp, the product size was 300bp, there were 16 methylation sites, and the GC content ranged from 60 to 64%. The target fragment in the DMR region of NR1D1 gene was 296–476bp, the product size was 181bp, there were 7 methylation sites, and the GC content ranged from 53.85 to 54.17%. The target fragment in the DMR region of RORB gene was 281–492bp, the product size was 212bp, there were 4 methylation sites, and the GC content ranged from 46.15 to 57.69%. The target fragment in the DMR region of MYOZ3 gene was 327–523bp, the product size was 197bp, there were 8 methylation sites, and the GC content ranged from 55.52–62.95% (Supplementary Figures S1–S5).

**Table 3:** Primers for amplicon of bisulfite sequencing PCR.

DMR	Gene	Primer sequence	Product Length
Chr20:43020501–43021000	*EDN1*	F: GGGTTTGTTTGAAGTTTTTTATTTA	273bp
		R: CCACTAAATCTCTTTACCTTCTTTTC	
Chr12:62293501–62294000	*LAMC2*	F: TTAGGTATTTTGGGGAAAGTTGTAG	300bp
		R: ACACACCACCTCCTCTATCTCTAAC	
Chr11:39923001–39923500	*NR1D1*	F: GAGGATGTGATATTTTAGGTAGTT	181bp
		R: CCATAATATTATCATTAAAAACAAAC	
Chr2:61615501–61616000	*RORB*	F: TGGATAGTTTTTTGTTGTAAGGATTG	212bp
		R: TCCAATATAAACCCAATCTCCTCTAT	
Chr5:59476501–59476600	*MYOZ3*	F: TGTTGAGTTATTGAAGGGTATTTTATT	197bp
		R: TTCCAAATCTCTCCCTAAACAAC	
Chr5:57479471–57480746	*WNT2*	F: GAAGTTATGTGTTGTGGTAGAGGTTA	230bp
		R: CCTTACAAATCCAATAAAATCCTTAC	

**Figure 5:**
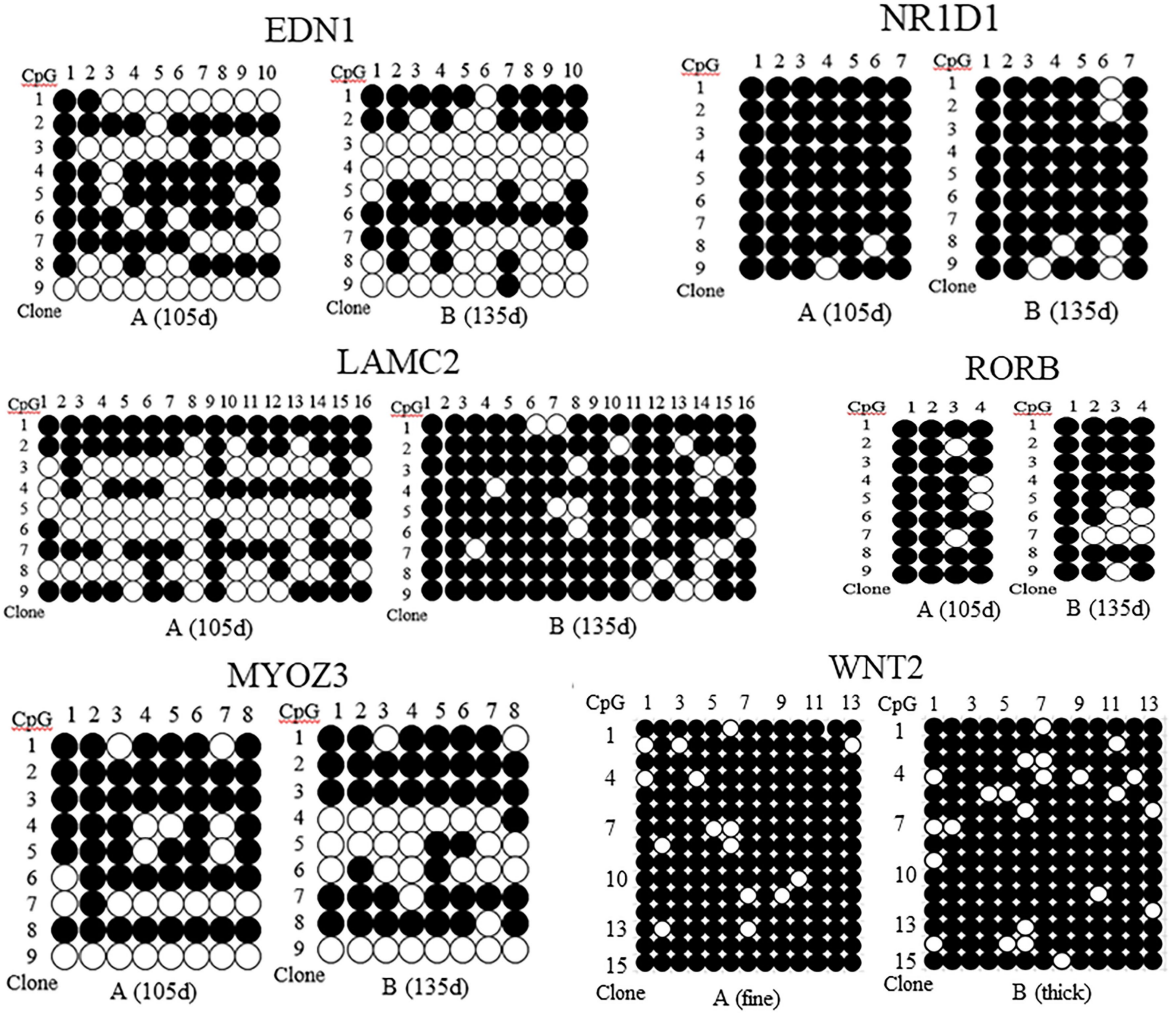
Methylation patterns of EDN1, NR1D1, LAMC2, RORB, MYOZ3, and WNT2 gene in skin tissues of Super Merino sheep with different stages and fineness. ●, indicates methylation; ○, indicates no methylation. **(A)** (fine), the extremely fine group; **(B)** (thick), the extremely thick group.

The methylation patterns of the sequencing genes corresponding to DMR were determined by sequence alignment analysis, and the methylation patterns of the five genes at 105d and 135d in the embryonic period were plotted. The results showed that the methylation rate of EDN1 gene was 54.44% at 105d and 42.22% at 135d in embryo. The methylation rate of LAMC2 gene was 52.78% at 105d and 85.43% at 135d. The methylation rate of NR1D1 gene was 96.83% at 105d and 90.48% at 135d. The methylation rate of RORB gene was 88.89% at 105d and 80.56% at 135d. The methylation rate of MYOZ3 gene was 66.06% at 105d and 56.94% at 135d (Figure 5). The methylation levels of EDN1, NR1D1, RORB, and MYOZ3 genes at 105d were higher than that of 135d at embryonic stage, while that of LAMC2 was the opposite.

**Figure 6:**
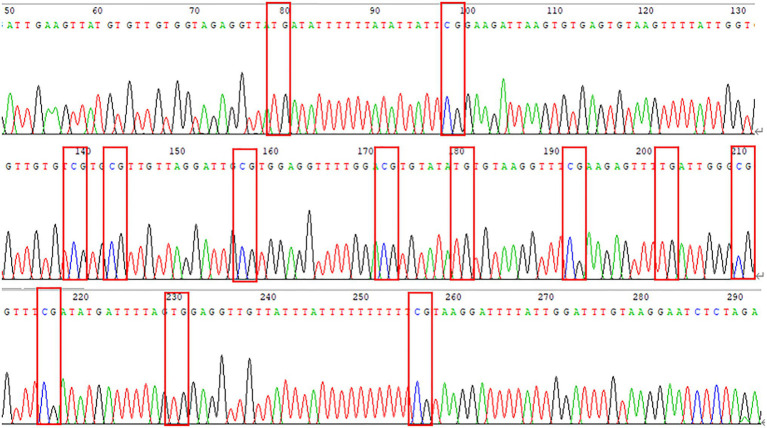
Peak sequencing of methylated region of *WNT2* gene.

The DNA methylation of the pre- and post-100bp of exon 5 of WNT2 gene in the skin tissue of Super Merino sheep was analyzed. The sequence alignment result found a total of 13 CpG sites were consistent with the results predicted by MethPrimer software (Figure 6). Three positive monoclones of WNT2 gene from the two groups (extremely fine group and extremely thick group) of Super Merino sheep were selected and sequenced. The DNA methylation patterns of WNT2 gene in the skin tissue of Super Merino sheep in two groups were represented by the black and white origin diagram. The methylation levels of the fine group and the thick group were both higher and the difference was not significant (*p*>0.05), and methylation ratios were 92.31 and 88.21%, respectively (Figure 5).

### Expression of DNMTs, EDN1, and WNT2 Genes in Sheep Skin Tissue

Gene sequences of DNMT1, DNMT3a, DNMT3b, EDN1, and WNT2 in sheep were provided according to the NCBI, and GAPDH was used as the internal reference gene. The primer sequences were shown in Table 4.

**Table 4:** PCR primers of qRT-PCR.

Gene	Primer sequence	Product Length/bp	Tm (°C)	GenBank No
*DNMT1*	F:CAGACGTGTGAGCCGAGTGAAC	117	61	NC_040256
	R:CGAGATGCCTGCTTGGTGGAAG			
*DNMT3a*	F:CGTCTCGGCTCCAGATGTTCTTC	180	60	NC_040254
	R:CGATGTAGCGGTCCACCTGAATG			
*DNMT3b*	F:TACCTCACCATCGACCTCACAGAC	94	60	NC_040264
	R:TGCTCTCCTGCTGGCTGTCC			
*EDN1*	F:GAAAGCCTGGGACAACCGAAAGAG	123	60	NC_040271
	R:TTGATGCTGTTGCTGATGGTCTCC			
*WNT2*	F:GAAGTTATGTGTTGTGGTAGAGGTTA	109	59	NC_040255
	R:CCTTACAAATCCAATAAAATCCTTAC			
*GAPDH*	F:GAGATCAAGAAGGTGGTGAAGCAG	113	60	NC_040254
	R:GTAGAAGAGTGAGTGTCGCTGTTG			

**Figure 7:**
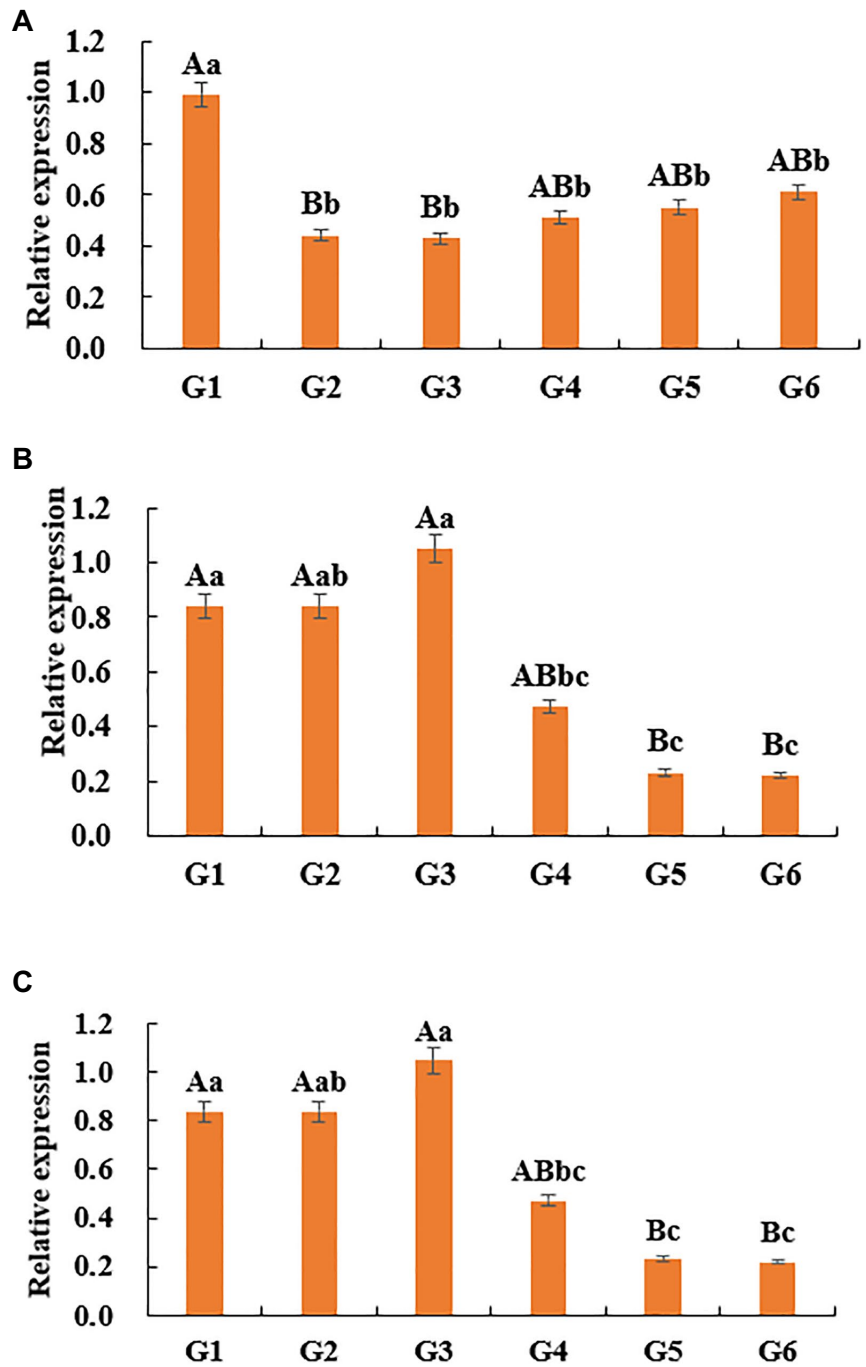
Relative expression of *DNMTs* genes in skin tissues of Super Merino sheep. **(A)** The relative expression of *DNMT1* gene. **(B)** The relative expression of *DNMT3a* gene. **(C)** The relative expression of *DNMT3b* gene. Different lowercase letters indicate significant difference (*p*<0.05), and different uppercase letters indicate extremely significant difference (*p* <0.01), the same letter expressed surprise was not significant (*p*>0.05).

The relative expression level of DNMT1 gene in G1 stage was significantly higher than that in G2 and G3 stages (*p*<0.01), and significantly higher than that in G4, G5, and G6 stages (*p*<0.05; Figure 7A). The change of DNMT3a gene expression increased first and then decreased, that is, it slowly increased from G1 period, reached the highest in G3 period, and then decreased. The expression level of this gene in G1, G2, and G3 was significantly higher than that in G5 and G6 (p<0.01), and the expression level in G1 and G3 was significantly higher than that in G4 (*p*<0.05; Figure 7B). With the hair follicle development, the expression pattern of DNMT3b in skin tissue showed a sequential pattern, that is, the trend of gradual decline. The expression level of this gene at G1 stage was significantly higher than that at G6 stage (p<0.05), and there was no significant difference between G2, G3, G4, G5, and G6 stages (*p*>0.05; Figure 7C).

**Figure 8:**
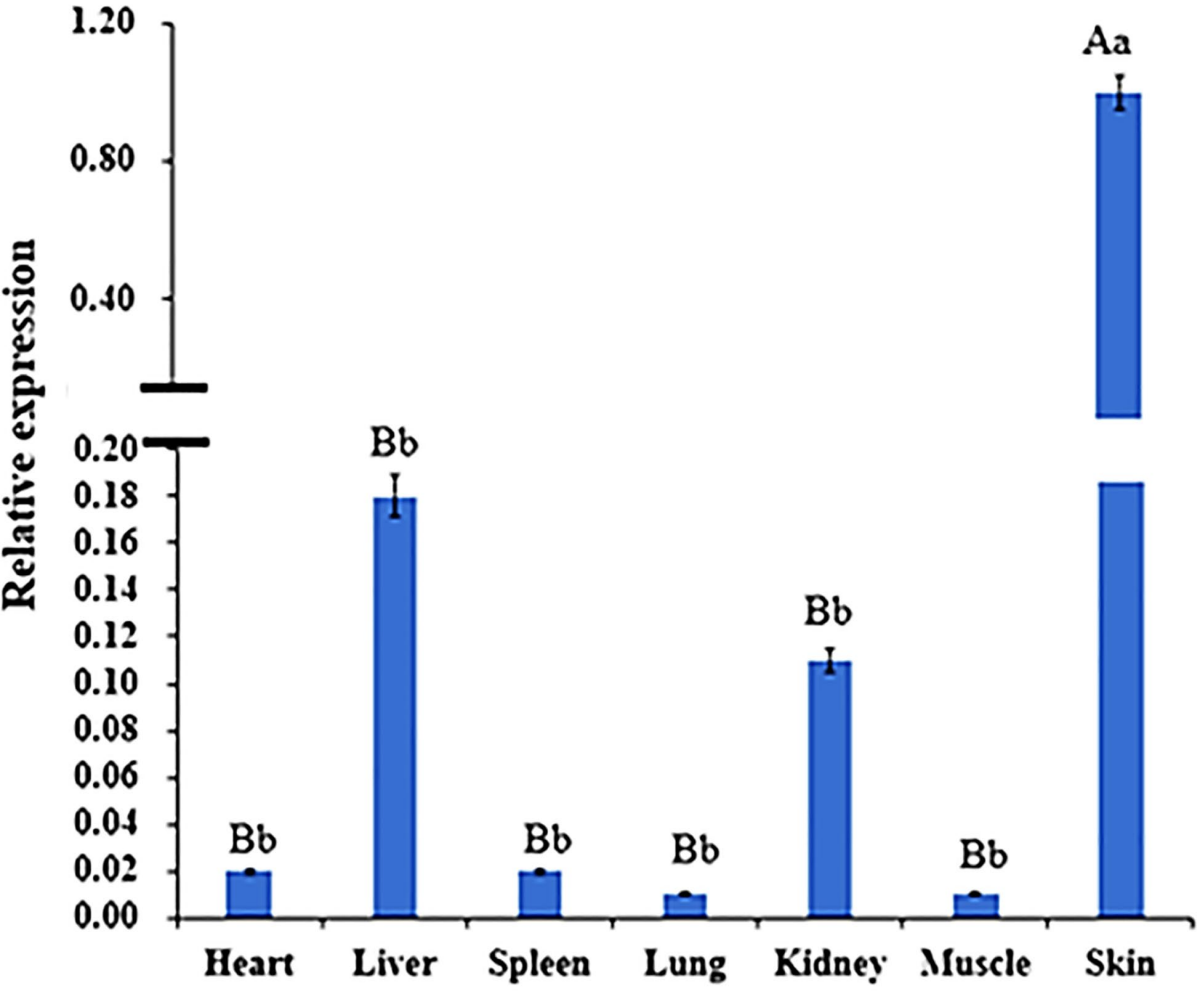
Expression of *WNT2* gene in different tissues at 135d of embryonic stage. Different lowercase letters indicate significant difference (*p* <0.05), and different uppercase letters indicate extremely significant difference (*p* <0.01), the same letter expressed surprise was not significant (*p*>0.05).

The results of WNT2 gene expression in different tissues at 135d in embryonic period showed that the ∆ct value of skin was the smallest, so skin was used as the control group to calculate the expression amount. WNT2 gene was expressed in all tissues, but the expression levels were different. The expression level of WNT2 gene in skin tissue was significantly higher than that in the other six tissues (p<0.01), and the expression level of WNT2 gene in heart, liver, spleen, lung, kidney, and muscle was not significantly different (p>0.05; Figure 8).

### Study on Relationship Between Methylation Expression and mRNA Expression in Super Merino Sheep

**Figure 9:**
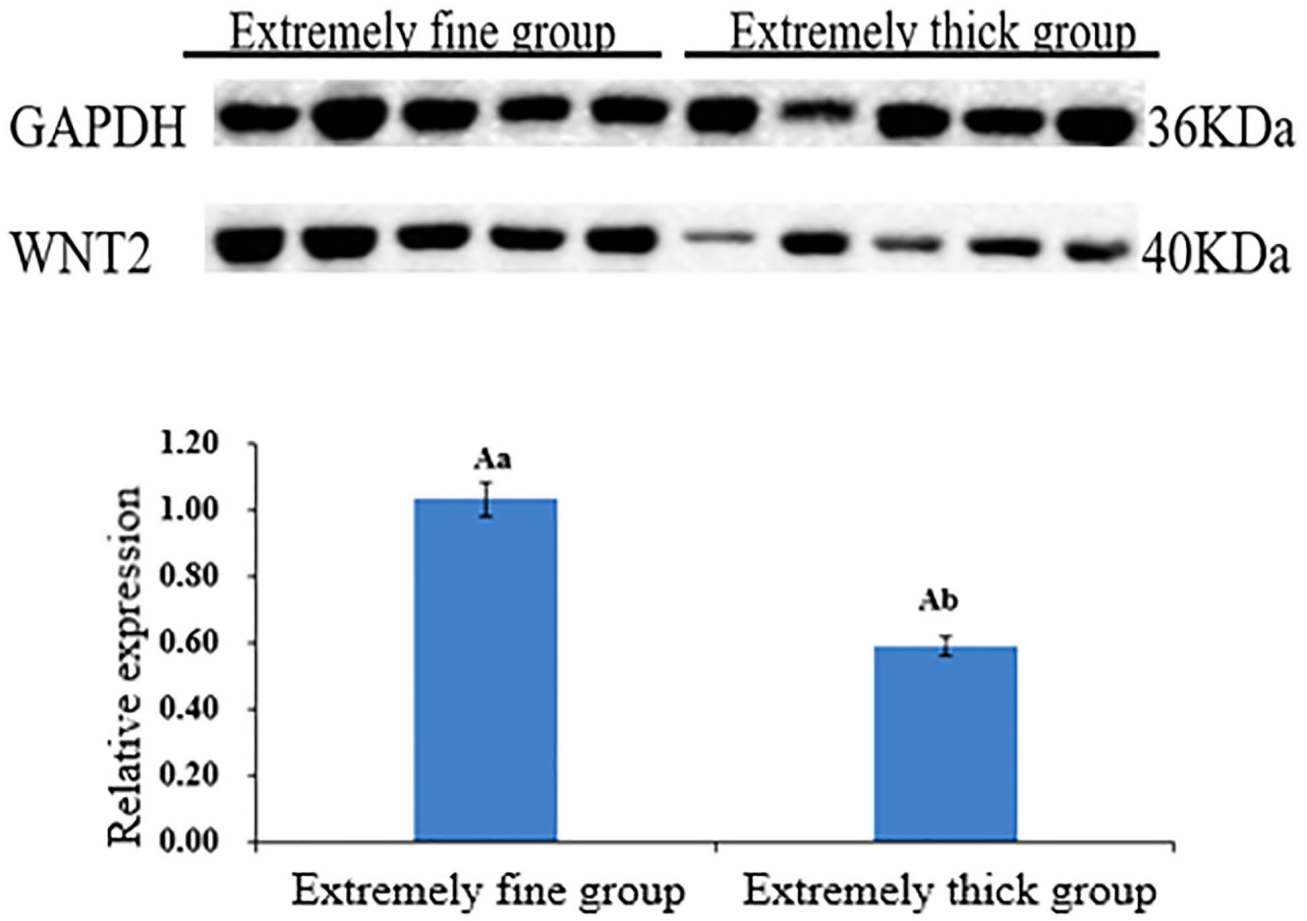
Relative expression levels of WNT2 protein in skin tissue of Super Merino sheep with different fineness. Different capital letters indicate significant differences (*p*<0.01), and same capital letters indicate no significant differences (*p*>0.01); different lowercase letters indicate significant differences (*p*<0.05).

Western Blot was used to detect the expression of WNT2 protein in the skin tissues of Super Merino sheep with different fineness, and it was found that WNT2 protein was expressed in each individual in the very fine group and the very thick group. According to gray value calculation (Figure 9), the expression level of WNT2 protein in the very fine group was 1.74 times higher than that in the very thick group (*p*<0.05), and the expression pattern of WNT2 protein in the two groups was consistent with the mRNA expression pattern and the change trend of DNA methylation level of WNT2 gene, both of which were higher in the very fine group than in the very thick group.

**Figure 10:**
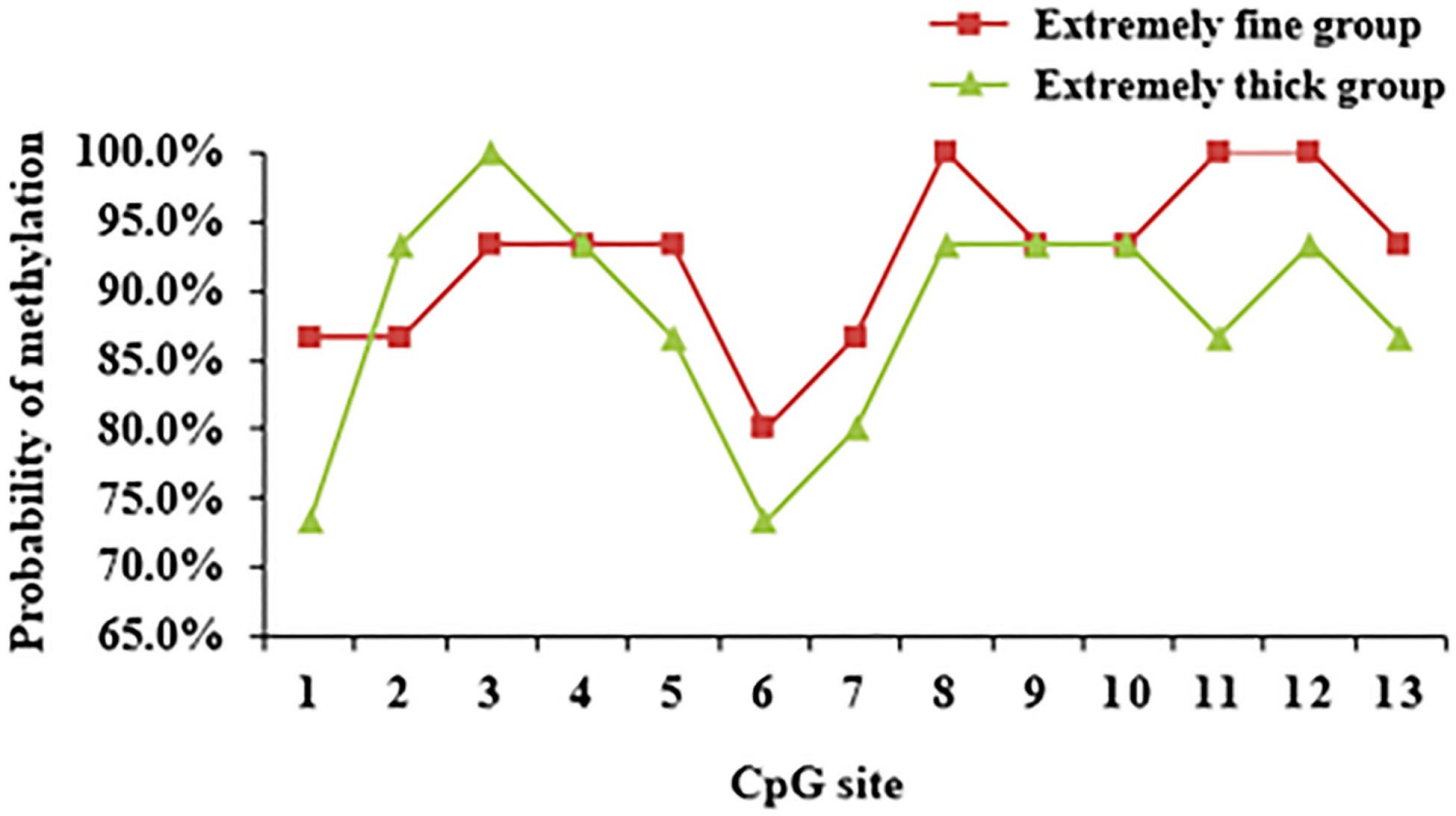
DNA methylation level of CpG loci of *WNT2* gene in skin tissue of Super Merino Sheep with different fineness.

Each CpG site of WNT2 gene had different methylation levels in the skin tissues of Super Merino sheep with different fineness, among which CpG11 site had the largest difference in methylation levels between the two groups (Figure 10). Correlation analysis was conducted on each CpG site and its expression level in the extremely fine group and the extremely thick group. As shown in Table 2, there was a significant positive correlation between the methylation level of CpG11 site and its mRNA expression level (*p*<0.05, r^2^=0.693; Table 5).

**Table 5:** Correlation between mRNA expression of *WNT2* gene and CpG site.

Relation expression	CpG1	CpG2	CpG3	CpG4	CpG5	CpG6	CpG7
	−0.228	−0.474	−0.265	0.036	0.098	0.128	0.450
	CpG8	CpG9	CpG10	CpG11	CpG12	CpG13	
	0.102	0.036	0.170	0.693^*^	0.313	0.353	

* *means significant correlation (p<0.05), unmarked means no correlation (p>0.05)*.

### Effect of WNT2 Gene on Proliferation of Sheep Skin Fibroblasts

The lentiviral vector pLEX-MCS was linked to the target fragment of WNT2 gene. The results of agarose gel electrophoresis were consistent with the results of the target fragment. Then, the recombinant plasmid pLEX-MCS-WNT2 was successfully constructed (Supplementary Figure S6). Sequencing was performed on the recombinant plasmid pLEX-MCS-WNT2 successfully identified by double enzyme digestion test (*XhoI*/*NotI*), and it was found that the recombinant plasmid pLEX-MCS-WNT2 successfully obtained the original sequence in the CDS region of WNT2 gene (Supplementary Figure S7).

**Figure 11:**
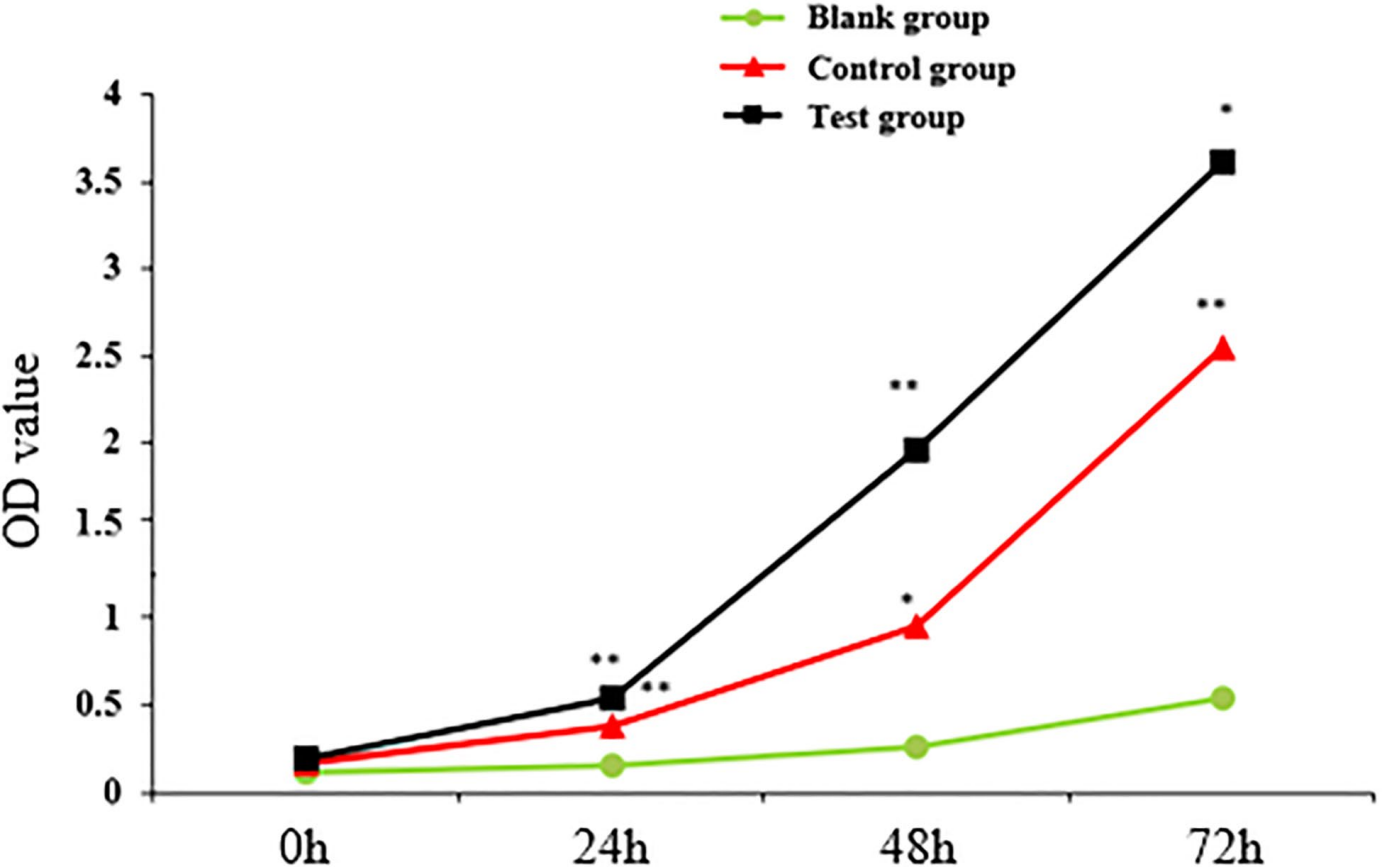
Detection of sheep skin fibroblast proliferation efficiency. ^*^ indicates significant difference (*p*<0.05), and ^**^ indicates extremely significant difference (*p*<0.01). Unmarked means the difference is not significant (*p*>0.05).

The proliferation of sheep skin fibroblasts transfected with lentivirus pLEX-MCS-WNT2 expression vector was detected by CCK-8 method. OD value represented the proliferation efficiency of sheep skin fibroblasts. With the increase of detection time, the proliferation level of sheep skin fibroblasts increased in blank group, control group, and experimental group, but the proliferation level in blank group was lower than that in the other two groups (Figure 11). At 24h and 48h. The proliferation level in experimental group was significantly higher than that in control group (*p*<0.01); the proliferation levels at 72h were significantly higher than those in the control group (p<0.05). The proliferation level of the control group at 24h was significantly higher than that of the blank group (p<0.01); the proliferation level at 48h was significantly higher than that in blank group (p<0.05); and the proliferation level at 72h was significantly higher than that in blank group (p<0.01). The results showed that WNT2 gene could promote the proliferation of sheep skin fibroblasts.

## Discussion

The quality of wool is determined by the structure and characteristics of hair follicles, so it is of great significance to improve wool quality to improve the regulation mechanism of hair follicle structure and development. Increasing functional molecules have been identified and characterized for each stage in hair follicle development of mice (Nakamura et al., 2013; Saxena et al., 2019; Zhao et al., 2020b). However, there are few reports regarding the machinery underlying fine sheep hair follicle morphogenesis due to technical difficulties and high costs. Although there are conservative signals in hair follicle development among mice, different physiology and regulation mechanisms exist between mice and sheep. Meanwhile, there are few studies on epigenetic markers and functional mechanisms related to hair follicle development in sheep. Taken together, further exploration and integration of the results of multi-omics studies are needed in order to better understand the regulation mechanism of hair follicle development.

The well-known genome-wide association study (GWAS) approach has been used to explain the variation in desired phenotypes among animals; however, in humans, the DNA sequence variation identified by GWAS is estimated to account for less than 30% of the phenotypic variation (Langevin and Kelsey, 2013). Therefore, in the current era of omics, epigenome-wide association studies (EWAS) may help to fill the gap between large-scale genomic information and related applications (Michels et al., 2013; Lee et al., 2014). Studies have shown that DNA methylation can lead to phenotypic changes in feeding and environmental conditions, resulting in productivity changes and animal disease risk (Boddicker et al., 2016; Tao et al., 2019; Tanwar et al., 2020). For example, a comparison of DNA methylation distribution between fast and slow growing broilers found that a total of 13,294 methylation genes were detected, of which 132 differentially methylated genes were related to growth and metabolism (Hu et al., 2013). Therefore, it is of great significance to explore the DNA methylation pattern of hair follicle development and the mechanism of candidate genes for improving wool quality breeding.

In this study, the MeDIP-seq technique was used to map the genome-wide methylation of skin tissue of Super Merino sheep during six stages of hair follicle development (Ablat et al., 2017). Results found 596 differentially methylated genes. Combined with the results of transcriptome sequencing, we found 65 genes that were both differentially methylated and differentially expressed at different stages of hair follicle development, including EDN1, LAMC2, NR1D1, RORB, MyOZ3, and WNT2 gene (Table 2). GO enrichment analysis showed that these differentially expressed genes were mainly distributed in biological processes, molecular functions, and cell composition. KEGG pathway enrichment analysis showed that most of the differentially methylated genes were distributed in metabolism, cancer, and some signaling pathways. Among them, Wnt/β-catenin, as a classical signaling pathway, was first found to be related to hair follicle development (Leirós et al., 2012). This indicated that the MeDIP sequencing results of this study are reliable. Meanwhile, comparison of dibisulfite sequencing results and MeDIP-seq results showed that the methylation levels of these differentially methylated regions were also basically consistent with the results of MeDIP-seq sequencing (Figure 5). It was proved that the MeDIP-seq data obtained in this study were reliable and could be used for the analysis of skin tissue of Super Merino sheep at different follicle development stages.

Considering the critical role of the Wnt signaling pathway in the development of hair follicles, as well as the result of MeDIP-seq, we want to further explore the methylation pattern and functional mechanism of WNT2 gene in the process of hair follicle development. Result showed WNT2 gene was widely expressed in the heart, liver, spleen, lung, kidney, muscle, and skin tissue of Super Merino sheep at 135d of embryonic period, and the expression level in the skin tissue was significantly higher than that in other tissues (*p*<0.05). It suggesting that WNT2 gene has an important effect on the growth and development of skin and hair follicle (Figure 8). The expression of mRNA and protein of WNT2 gene in the extremely fine group was significantly higher than that in the extremely thick group (*p*<0.05), which showed that WNT2 gene is a key factor regulating the early development of sheep hair follicles. In addition, the CpG11 methylation level of WNT2 gene was positively correlated with the mRNA expression (*p*<0.05), which indicated that the DNA methylation level of WNT2 gene promoted the expression of WNT2 gene, and then positively regulated the wool fiber diameter. This might be because the primer designed in this study selected the exon 5 of WNT2 gene, and it was the last exon of WNT2 gene. The influence of DNA methylation level in last exon region on the transcription level was greater than that in the promoter region, and the methylation of this region was positively correlated with gene expression (Yang et al., 2014; Singer et al., 2015). This is consistent with previous reports (Fraga et al., 2003; Zhang et al., 2006). These results showed the CpG11 site could be used as a candidate epigenetic marker (Table 5).

This study has verified that WNT2 gene was related to hair follicle development, and the DNA methylation level of WNT2 gene has a certain effect on wool fiber diameter. In order to further study, the regulation of WNT2 gene on hair follicle development, the lentivirus pLEX-MCS-WNT2 expression vector, was successfully constructed and transfected into sheep skin fibroblasts. It was found that WNT2 gene could promote the proliferation of sheep skin fibroblasts. It was further suggested that WNT2 gene could promote the growth and development of skin and hair follicles. According to previous study, Chen et al. (2018b) found that miR-125a can directly recognize and bind to the 3’UTR region of WNT2, and the regulation of WNT2 expression by miRNA may depend on transcriptional degradation. miR-125a also regulates hair follicle development by affecting the expression of WNT2-concordant genes in the Wnt signaling pathway, such as CTNNB1, LEF-1, PPARd, and TGFB1, so the relationship between miR-125a and WNT2 gene in the process of hair follicle development can be further explored. Studies also found androgens deregulate dermal papilla cells secreted factors involved in normal hair follicle stem cell differentiation *via* the inhibition of the canonical Wnt signaling pathway (Leirós et al., 2012). It was found that WNT2 is a Wntl protein involved in the classical Wnt/β-catenin pathway, and other WNT5a proteins involved in non-classical pathways. Studies have shown that classical pathways have a greater impact on induced hair follicle formation (Lin et al., 2015). Taken together, these results indicated that WNT2 gene may participate in hair follicle development through a variety of signaling ways, and its specific research is still worthy of further exploration.

## Conclusion

In this study, the MeDIP-seq technology was used to obtain the genome-wide methylation map in six stages of hair follicular development of Super Merino sheep. Combined with the results of previous transcriptome sequencing, 65 genes were screened out that were both differentially methylated and differentially expressed, including EDN1, LAMC2, NR1D1, RORB, MyOZ3, and WNT2 gene. Differential methylated genes were enriched in Wnt, TNF, TGF-Beta, and other signaling pathways related to hair follicle development. Further exploration of the function of WNT2 gene revealed that the DNA methylation level of its exon 5 promoted the expression of this gene. The overexpression vector of lentivirus pLEX-MCS-WNT2 was constructed, and WNT2 gene effectively promoted the proliferation of sheep skin fibroblasts. These results revealed the epigenetic regulation mechanism of hair follicle development and the regulation mechanism of WNT2 gene. It could promote the growth and development of skin and hair follicles and provide theoretical basis for sheep wool breeding.

## Data Availability Statement

The datasets presented in this study can be found in online repositories. The names of the repository/repositories and accession number(s) can be found at NCBI (accession: PRJNA750351 and PRJNA750354).

## Ethics Statement

The animal study was reviewed and approved by the Faculty Animal Policy and Welfare Committee of Xinjiang Academy of Animal Sciences under contract.

## Author Contributions

XH and KT designed the experiment, contributed to the revision of the manuscript, and assisted in revising the final version of the manuscript. YT, XY, and JD performed all the experiments. WZ, WW, and JD collected the sheep samples. YT prepared all the figures and tables and drafted the manuscript. All authors have read and approved the final manuscript.

## Funding

This research was funded by the Natural Science Foundation of Xinjiang Province (nos. 2019D01B07 and 2020D03016), the Special Fund for the National Natural Science Foundation of China (no. 31760655), the China Postdoctoral Science Foundation (no. 2017M623287), and the China Agriculture Research System of MOF and MARA (CARS-39). YT was supported by the Tianshan Elite Project in Xinjiang Province.

## Conflict of Interest

The authors declare that the research was conducted in the absence of any commercial or financial relationships that could be construed as a potential conflict of interest.

## Publisher’s Note

All claims expressed in this article are solely those of the authors and do not necessarily represent those of their affiliated organizations, or those of the publisher, the editors and the reviewers. Any product that may be evaluated in this article, or claim that may be made by its manufacturer, is not guaranteed or endorsed by the publisher.

## Supplementary Material

The Supplementary Material for this article can be found online at: https://www.frontiersin.org/articles/10.3389/fgene.2021.735827/full#supplementary-material
