# Supplementary material for: Differential Methylation and Transcriptome Integration Analysis Identified Differential Methylation Annotation Genes and Functional Research Related to Hair Follicle Development in Sheep
Source: Front Genet. 2021 Sep 30;12:735827. doi: 10.3389/fgene.2021.735827 (PMC8515899; doi:10.3389/fgene.2021.735827)
Supplement: Supplementary file 1 [file Data_Sheet_1.docx]

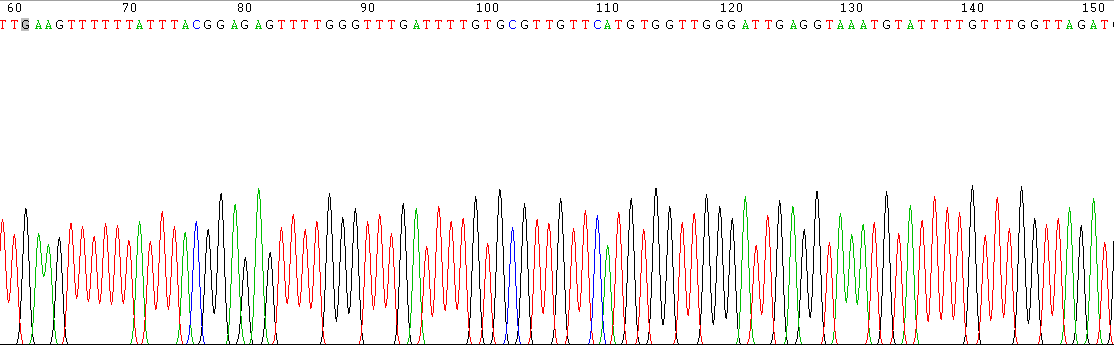

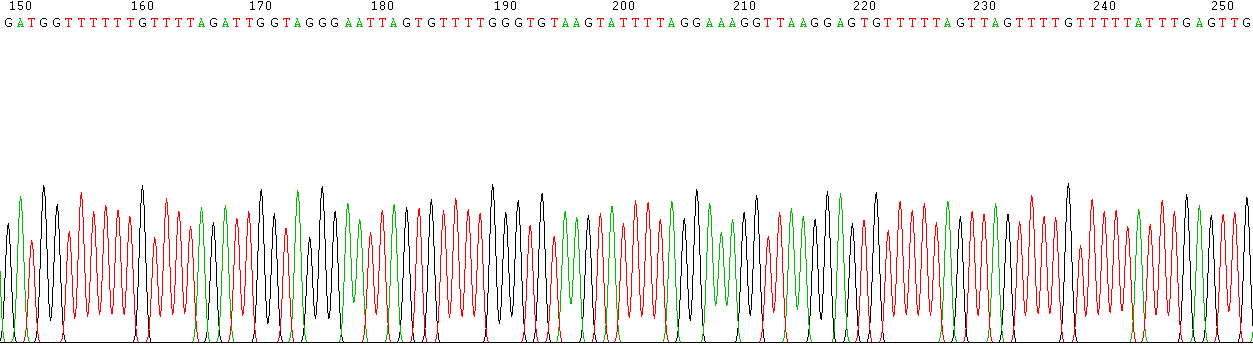

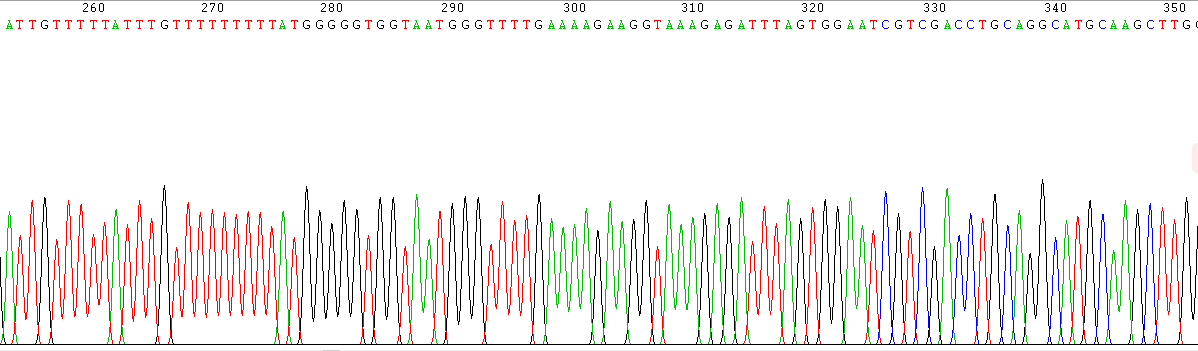


Figure S1. The bisulfite sequencing mode map for DMR in EDN1 gene


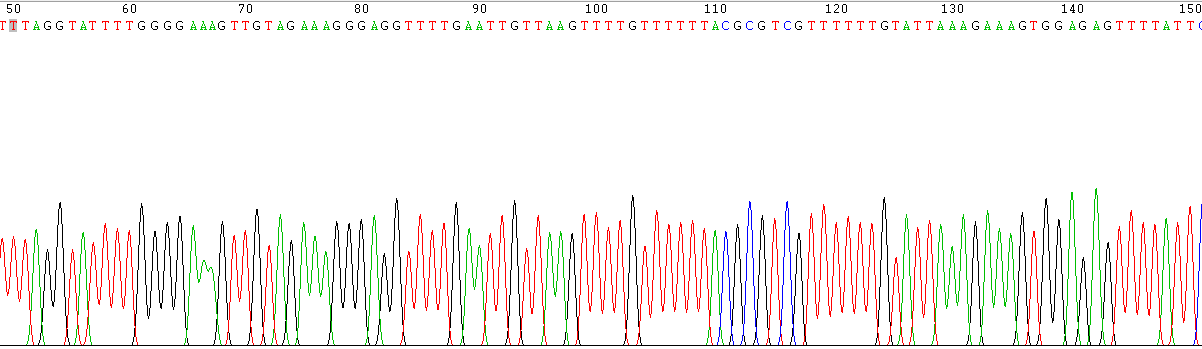

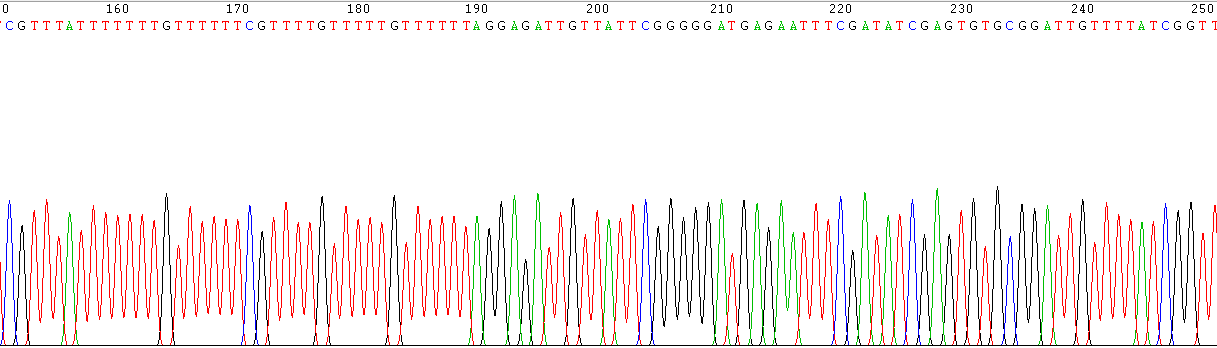

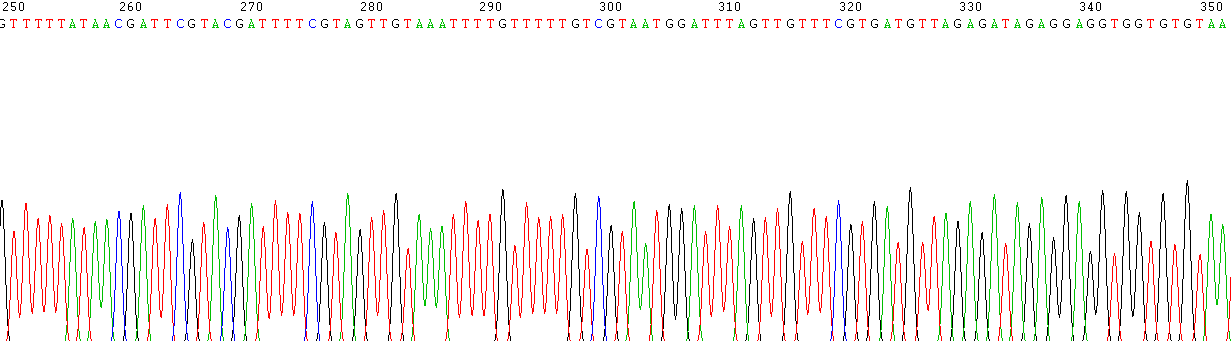


Figure S2. The bisulfite sequencing mode map for DMR in *LAMC2* gene

Figure S3. The bisulfite sequencing mode map for DMR in *NR1D1* gene


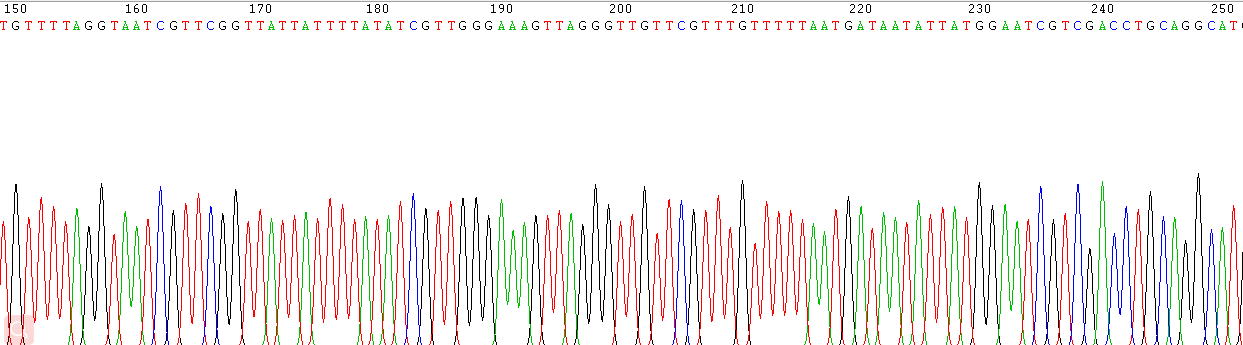

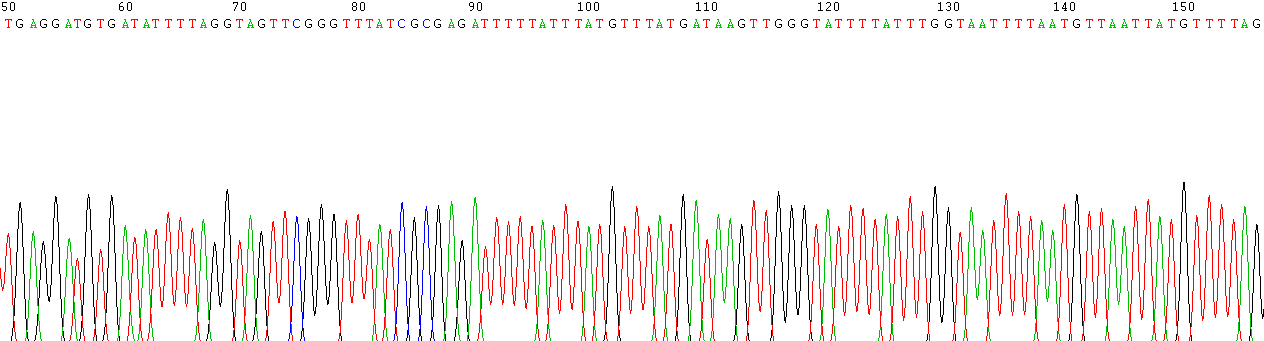

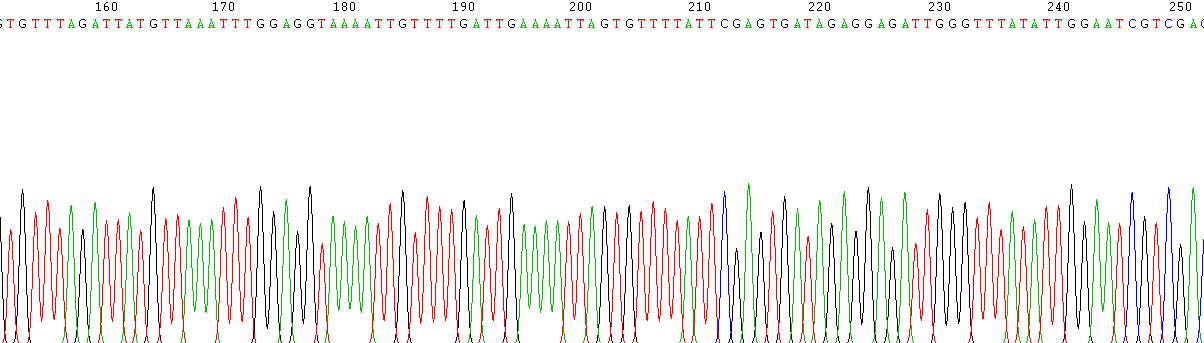

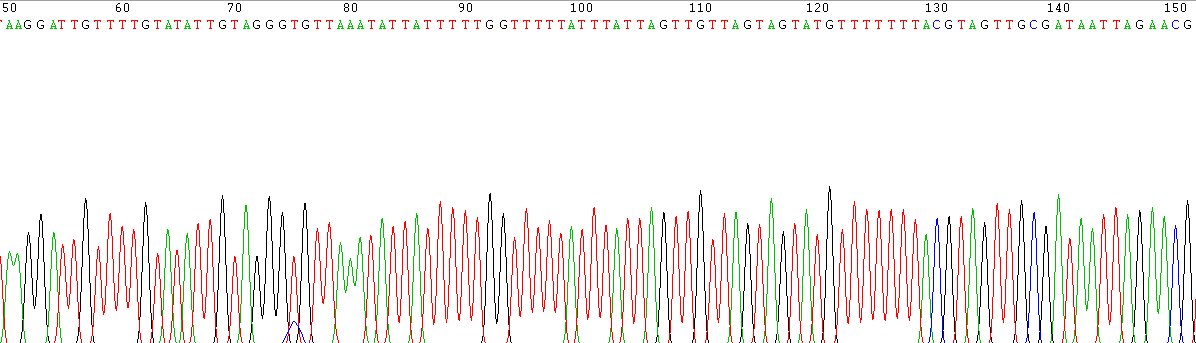


Figure S4. The bisulfite sequencing mode map for DMR in *RORB* gene


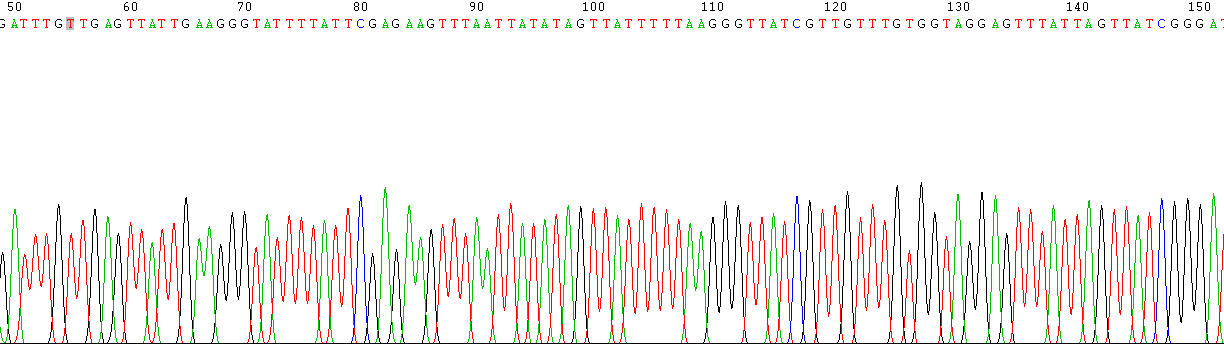

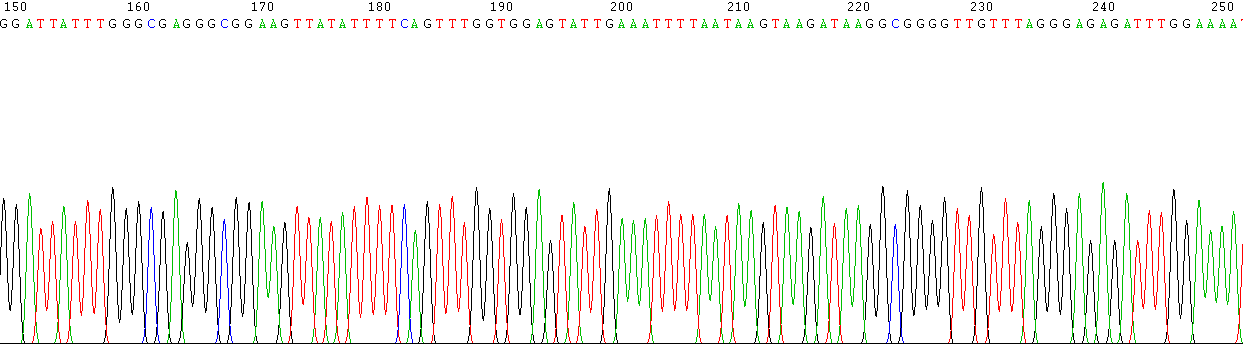


Figure S5. The bisulfite sequencing mode map for DMR in *MYOZ3* gene


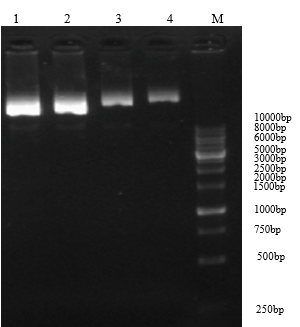


Figure S6. Extraction and identification of recombinant plasmid pLEX-MCS-WNT2

Note: No.1 and No.2 are empty lentiviral plasmids pLEX-MCS; No.3 and No.4 are recombinant plasmids pLEX-MCS-WNT2.


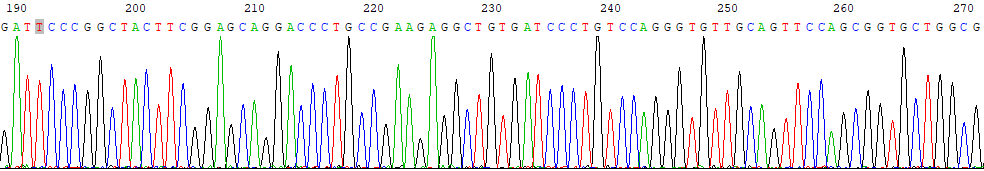

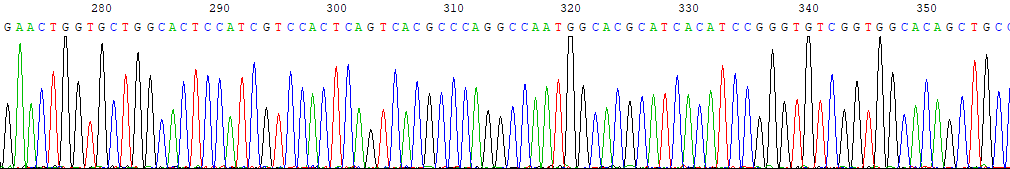

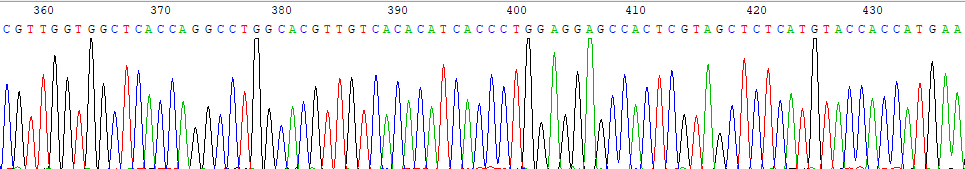

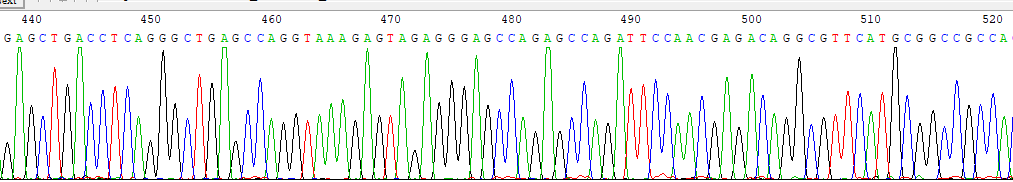


Figure S7. Peak sequencing of recombinant plasmid pLEX-MCS-WNT2
